# Development of a framework to improve the utilisation of malaria research for policy development in Malawi

**DOI:** 10.1186/s12961-017-0264-y

**Published:** 2017-11-21

**Authors:** Chikondi Mwendera, Christiaan de Jager, Herbert Longwe, Charles Hongoro, Kamija Phiri, Clifford M. Mutero

**Affiliations:** 1 University of Pretoria Institute for Sustainable Malaria Control (UP ISMC), School of Health Systems and Public Health, University of Pretoria, Private Bag X363, Pretoria, 0001 South Africa; 2 ICAP at Columbia University, Mailman School of Public Health, Pretoria, South Africa; 3 Population Health, Health Systems and Innovation, Human Sciences Research Council (HSRC), Pretoria, South Africa; 4 School of Public Health and Family Medicine, College of Medicine, University of Malawi, Blantyre, Malawi; 5 International Centre of Insect Physiology and Ecology (ICIPE), P.O. Box 30772, Nairobi, Kenya

**Keywords:** Malaria research, Research utilisation, Integrated knowledge translation, Research-to-policy frameworks, Malawi

## Abstract

**Background:**

The existing gap between research evidence and public health practice has attributed to the unmet Millennium Development Goals in Africa and consequently, has stimulated the development of frameworks to enhance knowledge translation. These efforts aim at maximising health research utilisation in policy and practice to address the world’s disease burdens, including malaria. This study aimed at developing a contextual framework to improve the utilisation of malaria research for policy development in Malawi.

**Methods:**

The study used two approaches including: two case studies of policy analysis exploring the policy-making process in Malawi, utilisation of local malaria research, and the role of key stakeholders in policy formulation process; and the assessment of facilitating factors and barriers to malaria research utilisation for policy-making in Malawi.

**Results:**

From the case studies’ lessons and elements identified during the assessment of facilitating factors and barriers, a framework is developed to promote an integrated approach to knowledge translation. In this framework the Ministry of Health is considered as the main user of knowledge from research through the demand created by the research directorate and the National Malaria Control Programme. Key documents identified as being particularly relevant to the Ministry of Health for purposes of knowledge translation include the National Health Research Agenda, Guidelines for Policy Development and Analysis, and Guidelines for Evidence Use in Policy-making. Institutions conducting academic and policy-relevant malaria research in Malawi are identified and a consolidation of their linkages with the users of research is established through the Knowledge Translation Unit, the Evidence Informed decision-making Centre, and the African Institute for Development Policy. Equally, key players in this framework are the funding partners for both research and programmes that need to see accountability and impact of their support. Independent advisors, partners, and consultants also have their vital role in the process.

**Conclusion:**

The framework offers a practical basis for the factors identified and their linkages to promote a co-ordinated approach to malaria research utilisation in policy-making. Its applicability and success hinges on its wider dissemination and ownership by the government through the National Malaria Control Programme.

## Background

Health research provides evidence that enhances knowledge, address health problems [1], and may potentially improve health systems, thus tackling the challenges faced by many developing countries such as Malawi [2]. However, the utilisation of research in addressing health issues has remained a challenge [3], prompting efforts of evidence-based practice that have mainly been exploited in the clinical set up leading to evidence-based medicine [4]. The concept of evidence-based medicine has progressively compelled non-clinical settings, including health policy, to utilise research evidence in policy development [5]. This has led to evidence-based policy-making, which urges policy-makers to demand and focus on using scientific evidence rather than political ideologies in policy decision-making [6, 7]. Despite this global drive, challenges of evidence utilisation in policy-making still exist, ranging from timely availability of evidence to the type of evidence itself [8]. This is further exacerbated by how the evidence is produced and the lack of proper channels of communication between researchers and policy-makers [9]. The promotion of evidence-based policy-making should not only focus on improving communication between policy-makers and researchers, but should take into consideration the diverse contextual factors influencing policy-making [8]. Assessment of these factors assists in developing contextual frameworks that embrace the facilitating factors and address the barriers to research utilisation in knowledge translation (KT). These frameworks, also referred to as models of research utilisation [10], are aimed at improving KT, which is described as “*the exchange, synthesis and ethically sound application of knowledge – within a complex system of interactions among researchers and users – to accelerate the capture of the benefits of research through improved health, more effective services and products, and a strengthened health care system*” [11–13]. The objective of KT extends beyond the dissemination of scientific information through publications as a primary form of spreading the research results. It involves all stages of the research process, interaction and engagement between the researchers and research users for the purposes of addressing the existing gap between large quantities of research evidence and its usage [14], and improving the lives of the general population [11, 15].

There are various frameworks promoting evidence-based medicine, but those that informed this study were based on the fact that their focus is in health policy formulation, they incorporate the stage of knowledge creation, promote the integrated model and consider the contextual factors in their application. These included the Canadian Institute of Health Research model of knowledge translation [16], the Knowledge-to-Action Process Framework [17], Tehran University of Medical Sciences Knowledge Translation Cycle [18], and the Ontario Drug Policy Research Network [19]. The first three are conceptual frameworks that provide an overall conceptual picture of how the knowledge translation process should occur. As conceptual frameworks, they do not highlight specific elements, such as individuals or institutions, nor their roles and responsibilities to facilitate particular processes in the framework. For an effective KT process it is important to take into consideration the contextual factors and micro-perspectives of individuals and institutions to support this process [20]. Therefore, further details showing comprehensive frameworks can augment these models. The Ontario Drug Policy Research Network organisational framework is an example of a detailed framework highlighting specific elements with their roles and responsibilities, arranged in a particular setting to boost interaction for the purposes of enhancing KT. This framework provides a practical perspective of how a contextual KT framework operates. Despite being a framework for commissioned research, it provides vital lessons on the interaction processes between researchers and policy-makers through research question formulation and the involvement of policy-makers throughout the research process, which facilitates the acceptability and utilisation of the research findings. These frameworks highlight the importance of KT in policy development, and the constant interaction between researchers and policy-makers in influencing this process.

In Malawi, malaria remains a major public health issue as it is estimated that approximately 4 million cases occur annually, primarily affecting children below the age of five and pregnant women [21]. Malaria research can play a vital role in addressing this burden by providing evidence for policy development leading to implementation of evidence-based interventions. However, the adoption of malaria research utilisation in policy development needs a systematic approach. Currently, no such approach exist in Malawi; thus, a framework to facilitate this process is paramount [10, 12]. This study presents the final product of a PhD research that aimed at developing a contextual framework to improve the utilisation of malaria research for policy development in Malawi. The specific objectives that contributed to the development of the framework include a determination of the type and amount of malaria research conducted and its related sources of funding from 1984 to 2016 in order to establish a malaria research repository, an exploration of the influence of malaria research on malaria policy development and a review of the policy-making process, and an assessment of the facilitating factors and barriers to malaria research utilisation for policy development, all of which were focused on Malawi.

## Methods

The development of the framework was based on lessons drawn from two case studies [22, 23] and an assessment of facilitating factors and barriers to malaria research utilisation in policy development in Malawi [24]. The methodologies for these studies can be accessed from the respective publications. In addition, basic concepts of research-to-policy frameworks were explored during the literature review and provided the underlying understanding of how frameworks are developed and how they operate [16–19]. The framework was finally exposed to a rigorous iterative approach with a sample of stakeholders for their views, validation and applicability.

## Results

### Lessons from case studies

Two case studies were conducted to examine the malaria policy development process and the contribution of research in this process.

#### Case study 1: Malaria research and its influence on antimalarial drug policy in Malawi

This case study, as fully described elsewhere [22], examined the influence of malaria research in changing the antimalarial drug policy in Malawi. Malawi changed its first-line anti-malaria drug treatment for uncomplicated malaria in 1993, from chloroquine (CQ) to sulfadoxine-pyrimethamine (SP), and later in 2007 from SP to lumefantrine-artemether. Since Malawi was the first country to switch from CQ to SP, many concerns were raised on the timing of the change and the early development of resistance of *Plasmodium falciparum* to SP. The case study examined whether the policy changes were justifiable by assessing the availability and utilisation of malaria research in this process. The study adopted a systematic literature search of published evidence of primary research from Malawi in the period between 1984 and 1993 when CQ was the first-line drug, and between 1994 and 2007 when SP was the first-line drug. In addition, relevant documents, such as malaria policy and guideline documents, were also reviewed and interviews were conducted with key informants involved in these policy changes.

The online systematic literature analysis included four publications during the period between 1984 and 1993, and four studies during the period between 1994 and 2007. Three studies during the period between 1984 and 1993 reported on poor efficacy of CQ, prompting policy change. The four studies identified between 1994 and 2007 were conducted in the early years of policy change and were aimed at monitoring the efficacy of SP. They all reported on the high efficacy of SP, of above 80%, and supported the use of SP as the first-line drug. However, towards the policy change in 2007, unpublished sentinel-site studies provided evidence that showed a reduction in SP efficacy, prompting a modification to lumefantrine-artemether. In addition, key informants acknowledged that both policy changes were justified based on local evidence.

This case study revealed how local evidence justified policy change amid the lack of WHO recommendations in 1984, yet the change in 2007 was smooth due to availability of WHO recommendations.

This case provided critical lessons for the framework by informing on the importance of generating local evidence in developing local policies, which may form the basis for decision-making despite unavailability of WHO recommendations. This evidence can be fully utilised with the government’s determination as demonstrated in the case study, whereby the government, through the National Malaria Control Programme (NMCP), commissioned studies to provide the evidence. This established that research has a high probability of being utilised if the demand is driven by the users (policy-makers). Therefore, the proposed framework emphasises the need for researchers to engage with policy-makers by exploring their research needs.

#### Case study 2: Changing the policy for intermittent preventive treatment with SP during pregnancy in Malawi

This case study, also described elsewhere [23], examined the policy change process of intermittent preventive treatment of malaria during pregnancy with SP (IPTp-SP) from the administration of two doses to three or more during pregnancy. Malawi was the first country to adopt IPTp-SP in 1993, whereby pregnant women were recommended to receive two SP doses during pregnancy. The growing resistance of *P. falciparum* to SP led to the change in treatment of uncomplicated malaria from SP to lumefantrine-artemether in 2007 and similar concerns were raised in the use of SP for IPTp amid a global lack of an alternative drug. In 2013, the IPTp policy was changed to recommend that pregnant women should receive at least three SP doses. The process of changing this IPTp-SP policy was assessed to gain an insight in the policy formulation process and the involvement of stakeholders and local research.

A mixed methods approach was adopted by an online systematic literature review, relevant documents assessment and key informant interviews. The online search reviewed eight studies from Malawi. Two publications were instrumental in changing the WHO IPTp-SP policy, which later made a recommendation for national policies to adopt the new policy of administering IPTp-SP at each antenatal visit with the first dose given as early as possible in the second trimester and the following doses given at monthly intervals up to the time of delivery. Malawi utilised this opportunity to adapt its IPTp-SP policy in 2013 to address the operational challenge during the implementation of the first policy of two SP doses. It was recommended that women should receive at least three SP doses during pregnancy with the last dose given close to birth and health workers were no longer confused with the timing of administering the doses.

The policy change revealed that malaria research from Malawi was instrumental in guiding policy change at the global level, but Malawi only changed its IPTp-SP policy following a WHO recommendation. However, it was highlighted that it is vital for the responsible government department to fully commit to driving the policy change and involve the relevant key stakeholders. The importance of local evidence was identified to be critical for policy decision-making and thus it was recommended that a systematic approach should be adopted to utilise evidence in developing local policies. Therefore, a malaria research-to-policy framework is ideal in addressing this challenge.

The case study has shown that malaria research conducted in Malawi is capable of influencing global policies and thus development of local policies should fully utilise this evidence in their development. This should also motivate local researchers to conduct rigorous research for policy change purposes. In addition, local evidence can assist in adapting WHO recommendations to suit the local context, while the inclusion of relevant stakeholders during policy change is critical. These lessons feed in the framework development as it is emphasised that local evidence is vital and a thorough stakeholder analysis is required before embarking on policy change.

#### Assessment of facilitating factors and barriers to malaria research evidence for policy development in Malawi

Utilisation of research evidence in policy formulation has not been straight forward; hence, research-to-policy frameworks have been developed for this purpose, although most of them have been in developed countries [10]. Consideration of contextual factors is essential in developing these frameworks [25], and Logan and Graham [12], who developed the Ottawa Model of Research Use, developed guidelines of developing contextual research-to-use frameworks for the improvement of health services. The basic approach involves the assessment of enablers and barriers in the utilisation of research evidence in policy. Therefore, an assessment of facilitating factors and barriers to malaria research utilisation for policy development in Malawi was conducted, the process for which is fully described elsewhere [24].

Drawing from the approaches above, we developed a framework appropriate to Malawi as it identifies specific elements or institutions, with their roles and responsibilities, and propose how they should interact to actively promote malaria research for policy development.

## Discussion

### The Framework

**Fig. 1:**
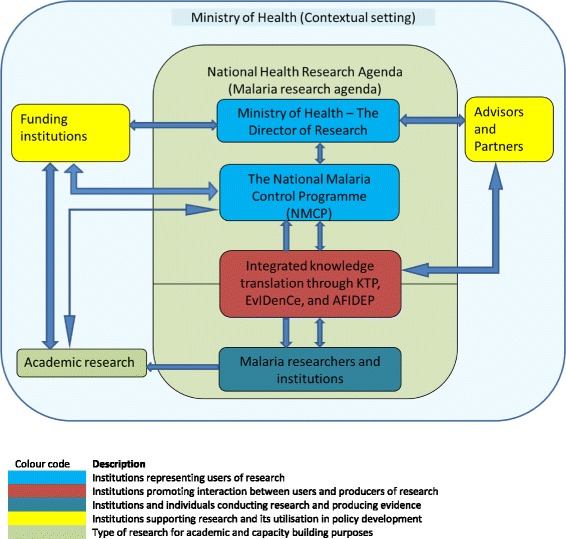
Framework to promote the utilisation of malaria research for policy development in Malawi

The framework is designed to provide rapid-response research for policy-making, which means that there is a unique blend between researchers and policy-makers reflecting the principles of the integrated model with the aim of providing timely, high-quality, policy-relevant research findings. The elements in the framework were identified in the assessment of facilitating factors and barriers, while lessons from the case studies have provided an insight on how the elements can interact. The assessment revealed the existence of elements promoting the utilisation of health research for policy formulation in Malawi, but the main challenge is the lack of a coordinated approach since they are fragmented and work in isolation while duplicating activities. The framework should thus enhance visibility and strengthen the interactions and coordination among these existing initiatives. A wider dissemination of the framework is paramount to serve this purpose. The interactions of elements are flexible and can occur in parallel. While the identification of elements is not exhaustive, the framework serves as a guide for new elements to recognise where to fit or with whom to engage in order to prevent duplication of activities. Figure 1 shows the structural set up of the framework, proposing feasible interactions among various elements with the purpose of promoting policy-relevant research in malaria. The roles and responsibilities of the elements are described below.

### Ministry of Health (MoH) - Contextual setting

The contextual setting is the environment in which the elements exist and whose conduciveness determines the successful interaction of the element in the framework. The contextual setting consists of the political set up, the leadership system within the MoH, government policies and the cultural set up. Malawi adopted a democratic government system in 1994, which has improved independent decision-making within institutions; this is one of the enhancing factors within the MoH. While advocating for research utilisation in the health sector, the MoH revived the Policy Development Unit and developed the guidelines for policy development and analysis, and for evidence use in policy-making. Another strategic element identified during the study was the quality of personnel entrusted in management positions within the MoH. The positions of Chief of Health Services, Principal Secretary, and Director of Research were all managed by enthusiastic individuals with doctorate qualifications of a medical or public health background, which makes them value the importance of using research in decision-making. Recognising and supporting research emanating from the MoH encourages the usage of research for policy development. The main challenge is the politically motivated job rotations/appointments that occur in government institutions, where newly appointed employees may not be as motivated or qualified to pursue the initiatives they inherit.

### The National Health Research Agenda (NHRA) – Malaria research agenda

The Malawi government recognised the important role of research in development. The main challenge for the country is that most of this research is funded externally by institutions who may drive their own agenda. It is against this background that Malawi developed its first NHRA covering a 5 year period from 2012 to 2016, and currently under review for a subsequent 5 year period of 2017 to 2022. The NHRA aims at guiding research conducted to address the country’s health needs.

The malaria research agenda, as part of the NHRA, forms the background of the institutional set up in the framework. The main purpose of the agenda is to guide researchers, policy-makers, health development partners, and other stakeholders on malaria research priorities for Malawi. The agenda outlines relevant research areas needing evidence for policy development.

### The Director of Research in the MoH

The MoH is responsible for health services delivery in the country and, therefore, it needs to promote health interventions with a proven track record. A key to this initiative was the establishment of the office of the Director of Research in the MoH. This office, which campaigns for policy-relevant research steered by the guidelines for evidence use in policy-making, is also the secretariat for the National Health Sciences Research Committee, a national health research ethical review board. It is thus tactical for the Director of Research to enhance the timely ethical review process of protocols addressing the NHRA with the aim of providing rapid-response research for policy development. However, an objective approach should be encouraged to avoid conflicts of interest.

### The NMCP

In order to create a high probability of research adoption in policy development, it is important that the drive for research emanates from policy-makers. The NMCP is the primary coordinator of malaria interventions in the country under the Directorate of Preventive Health Services in the MoH. Its mission is to reduce the malaria burden to a level of no public significance; hence, it is vital that tangible evidence is used when developing its policies. One of the critical sections of the NMCP is the monitoring and evaluation department, which provides routine evidence by assessing population-level information from the national health databases. It is a key department for the timely detection and response to research requests for policy decision-making. It will identify areas in malaria that require further understanding through research. However, the quality of population-level information depends on the quality of the national datasets, which have challenges originating from the sources at district levels. If poor data capturing is made at the district level, it becomes a challenge to rectify at the central level, leading to a decision or policy development based on inadequate, incomplete or poor quality evidence. Another important initiative at NMCP is the setting up of Technical Working Groups, which enable informal sharing of evidence and debate between researchers and the NMCP. In this regard, there is a continuous interaction between the two parties.

### The Knowledge Translation Platform (KTP), Evidence Informed Decision-making Centre (EvIDenCe), and African Institute for Development Policy (AFIDEP)

These three KT institutions are vital in promoting the integrated model that emphasises the involvement, at an equal level, between researchers and policy-makers to develop and conduct relevant research that is likely to be used [26]. They are tasked with making all efforts and strengthening communication, which has been identified as one of the challenges in evidence-based policy development [9]. Their interaction is critical to prevent the duplication of roles. Further, their establishment signifies the importance that Malawi has placed on exploiting research evidence for decision-making and policy development.

#### The KTP

Housed under the Director of Research, its key mission is to provide an environment through which researchers, policy-makers and stakeholders can discuss essential local or international research findings to increase the relevance and contribution of research to high-priority issues in Malawi. The KTP specifically aims at identifying high-impact policy issues in relation to established national priorities for which primary research and other evidence-based inputs are required, coordinating efforts to use timely local and international evidence in policy-making through policy dialogues and inputs such as policy briefs, reviews, publications and reports, and initiating and facilitating opportunities for researchers, policy-makers and stakeholders to build their capacity to use evidence in policy-making.

The KTP serves the entire MoH and is thus tactical for NMCP to engage with the KTP in addressing issues specific to malaria. Housing the KTP under the MoH is advantageous when accessing information since the MoH commands some greater authority than if it were independent.

#### The EvIDenCe

The EvIDenCe is the first, recently established, academic unit in the country to promote evidence-based health practice and economic evaluation. Based at the College of Medicine, a constituent college of the University of Malawi, this provides an opportunity to fill a key gap in research capacity, health economics and evidence synthesis that could inform policy formulation as well as practice. Its main purpose is to strengthen translation of research into policy through conducting and teaching systematic reviews, evidence-based healthcare, evidence synthesis and development of health research databases to update the health research activity in Malawi. It also undertakes other tasks such as the renewal of the NHRA and carrying out such research in collaboration with capable institutions or individuals. Since academic institutions are recognised to passively disseminate their research mainly through publications, which are insufficient to guarantee adoption by policy-makers [27], the EvIDenCe is responsible for synthesising the research findings and disseminating these to the relevant stakeholders through the use of dissemination tools such as policy briefs.

#### AFIDEP

As an independent organisation, AFIDEP complements the work by KTP and EvIDenCe as it focuses on capacity strengthening and knowledge synthesis, translation, and utilisation. Through the Strengthening Capacity to Use Research Evidence in Health Policy programme, AFIDEP strengthens the capacity of health policy-makers and legislators in research evidence utilisation for decision-making. Their aim is to consolidate interaction of researchers and policy-makers and thus improve on their mutual trust. One of its specific activities of interest is building the capacity of policy-makers to access, appraise and apply research evidence in their decision-making and policy development. This initiative is vital in instilling a culture of evidence use for decision-making among policy-makers.

### Malaria research institutions

This block constitutes various institutions and individuals that conduct malaria research in Malawi. International institutions and individuals should access the NHRA to familiarise themselves with the country’s priority areas of health research. Notably, among others, the major malaria research institutions in Malawi are the University of North Carolina project, the MoH and the College of Medicine, whose affiliates are the Malaria Alert Centre, Malawi-Liverpool-Wellcome Trust and Blantyre Malaria Project. The Malaria Alert Centre conducts both basic medical and operational research and, in partnership with the NMCP, responds to research needs relevant for national health policy development, while the University of North Carolina, Malawi-Liverpool-Wellcome Trust and Blantyre Malaria Project mainly conduct clinical malaria research with the aim of contributing to evidence-based malaria policies and capacity-building in the country.

The advantage that these institutions have in conducting malaria research is their financial support, research capacity and infrastructure to support quality research. Another vital feature is the existence of the College of Medicine Research and Ethics Committee, which conducts ethical reviews for these research institutions, reducing the pressure on the National Health Sciences Research Committee and making it convenient for academic research to be timely conducted.

### Academic research

The NHRA highlights priority policy-relevant research needs in Malawi. However, researchers can also conduct other types of research for academic purposes, which can play a vital role in providing evidence that can be used at a later stage while strengthening the capacity of researchers to eventually conduct quality policy-relevant research. In this respect, researchers are engaged in impactful academic research independent of the malaria research agenda needs, which can also be shared with the NMCP for their reference.

### Advisors and partners

These institutions provide advice, consultation and work in partnership with the MoH in either supporting the ministry in policy and guideline development or training of policy-makers in the ministry to view research evidence as vital for policy decision-making. These institutions can collaborate with the KTU, EvIDenCe and AFIDEP to refine messages to be communicated to policy-makers and to increase the general community’s understanding of research findings. WHO plays the advisory role of ensuring that the health policies implemented are in accordance with WHO guidelines. However, WHO will not impose policies, but will rather provide guidance based on global research for countries to adopt or adapt these based on their context. It is therefore the responsibility of the KTU, EvIDenCe and AFIDEP to assess the evidence on which WHO policies are based in comparison to local evidence if available and advise the NMCP on the way forward.

Another prominent partner working with the MoH identified during the time of the study was the Support for Service Delivery Integration, a consortium of three projects based on services, systems and communications. Through this, the systems project, led by Abt Associates, is aimed at improving the health system in Malawi by building capacity for policy development, strengthening capacity and leadership, assisting the MoH in the development of evidence-based policies, and improving the usage of routine health information.

### Funding institutions

Funding institutions comprise research and programme funders. Challenges of research funding have always existed in Malawi, hence its commitment to the establishment of the National Commission for Science and Technology to play an advisory role to government and stakeholders on matters of development, science and technology. One of the schemes is the provision of small research grants supporting studies addressing the NHRA. However, these grants are not adequate for larger studies, prompting the government to liaise with other independent research funding institutions and the need for a wider dissemination of the NHRA. Some of the main funding institutions supporting malaria research and programmes are the United States President’s Malaria Initiative, the Global Fund, WHO, UNICEF and USAID, which may have a greater bearing on how their funds are used and can play a critical role in advocating for their funded research to impact on policy and practice [28].

### Application of the framework

The research-to-policy process is complex, with no precise fitting blueprints [10]. Various frameworks exist; however, most are conceptual, explaining the process of KT and assume that the contexts in which they are applied are uniform, ignoring the intricacy of specific environmental factors [10]. Thus, this framework dwells on the identification of specific contextual elements to augment the process of KT while utilising the concept of dynamic multi-directional processes, which recognises that KT is a function of multiple stakeholders’ collaborations and interactions that can occur simultaneously.

As described above, the framework has highlighted the existing elements promoting utilisation of research for policy development in Malawi. These elements have thus been organised to promote this process. The guiding principle in the structural set up of the framework is to promote the integrated KT model.

The integrated approach to KT seeks to bring together knowledge users and researchers to commonly pursue health challenges and find solutions together. Knowledge users are described as “*individuals who are likely to be able to use research results to make informed decisions about health policies, programs and/or practices*” [26]. Understanding the various knowledge users is critical in adopting strategies to engage them in the research process. Depending on the research focus, users can include, among others, policy-makers, programme managers, clinicians, health-related training lecturers and patients or the public itself [29]. In consideration to contextual factors, including the research focus, knowledge users can be engaged at various stages in the research process, which can include research question identification, definition and development, conducting research, and interpretation and application of research findings [30]. The targeted knowledge users in this framework are policy-makers in the MoH through the NMCP. The malaria research agenda is the guiding principle, and its development provides the initial stage of interaction between researchers and policy-makers. It is through a thorough involvement of the two parties that a viable relationship can emerge, promoting participatory research. Since the agenda provides broad areas of research needs, researchers must formulate their research questions based on these areas and engage the NMCP for a common understanding. This engagement is aimed at refining the objectives of the research and confirming its feasibility while developing timelines, in order to confirm that the research focusses on the needs of the NMCP. It is important to seek the approval of the NMCP if the intention of the findings from the research is to have a bearing on policy. Therefore, despite the researcher’s effort in securing funding, the research must be representative and conducted in a manner in which the NMCP can utilise the findings.

In addition, the Technical Working Groups at the NMCP are opportunities in which a continued relationship is established, where the NMCP can express further research needs while researchers can update the NMCP on various stages of the research process. In this way, both policy-makers and researchers are aware of the available research evidence and needs, respectively, thus increasing the relevance and utilisation of research findings. Once the research has been conducted, researchers and policy-makers can further be engaged through the KTP, EvIDenCe and AFIDEP to package and communicate the research findings in an appropriate format. However, researchers are also encouraged to publish the research findings for the wider scientific audience, which will also serve well for their academic advancement.

From the researchers’ recommendations, a policy position must be established while highlighting alternative options. It is at this level that researchers are required to understand that their research can be used for different purposes during policy development. The findings can be instrumental if they directly lead to policy change, conceptual if they are used gradually as theories, concepts and perceptions, and symbolic if they support an already known policy stand [10]. In addition, the findings can be used either during policy agenda setting, policy formulation or policy implementation stages as identified by Lavis et al. [31]. This requires documentation to track how the research evidence was utilised in this process. However, the evidence-to-policy development process can be enhanced through the researcher–policymaker model [32]. Therefore, collaboration and understanding between researchers and policy-makers is vital in facilitating usage of evidence in policy-making and to address the knowledge-to-action gap affecting health systems globally with KT labelled as the dominant problem [29]. The KT paradigm has predominantly assumed a unilateral approach, in which either researchers conduct research, mostly in isolation, and seek the best approach to disseminate the findings to passive research users such as policy-makers (the push model), or research users seek for evidence or commission research to be conducted (the pull model) [29]. This has emphasised the need for knowledge brokers to focus on finding the best approaches, requiring skills and resources, in KT [33]. However, dissemination of research findings alone has limited impact despite using creative approaches [34]. Therefore, a participatory approach engaging users of research in the research process has predicted a high use of research findings [35].

The NMCP’s role is pivotal in this framework because it is responsible for developing malaria programmes implemented in the country. It is accountable in the development and implementation of interventions that work, which can only be identified through research. The NMCP will strive to access research evidence for its planning and hence develop research questions and work with researchers. Therefore, the NMCP should be custodian of this framework, which should be included in the malaria research agenda, in guidelines for policy development and analysis, and in evidence use in policy-making for the purpose of its formal institutionalisation and wider dissemination.

Being a results-oriented institution, the NMCP should make all efforts to facilitate the conduct of policy-relevant research and its uptake for policy development. The NMCP has already shown that engaging researchers at various stages of the research process is important. For example, its unique partnership between the NMCP and the Malaria Alert Centre in facilitating research that provides evidence for the programme to utilise, is important because research commissioned by the users has greater likelihood of being used for policy development [36]. Similar arrangements exist between the mental health research unit and the mental health reform branch of the Ontario government, which make it strategic to commission research specific for policy and programme development [37].

Understanding each institution’s role and collaboration during the research process between researchers and policy-makers (NMCP) can lead into an equally positive partnership. This collaboration should start at the stage of malaria research agenda development and should be further consolidated through the research process up to utilisation. If these collaborations are maintained, a rapid-research process should be established and enhanced, leading to timely availability of research findings for policy development. This incorporation of research into the policy-making process should result in informed decisions that positively impact on the health of the communities.

## Conclusion

The framework identifies specific elements or institutions that should be actively involved in malaria research for policy development and their linkages to promote a co-ordinated and integrated approach to KT. Its applicability and success hinges on its wider dissemination and ownership by the government through the NMCP.

This framework will be useful to researchers conducting non-commissioned research as it provides direction if they intend to influence malaria policy in Malawi. In addition, it will guide policy-makers on the procedures to be followed when seeking evidence for policy development. The framework further offers a visual presentation of elements involved in the research-to-policy process, hence bringing visibility and coordination in their roles and responsibilities.

## Abbreviations

AFIDEP: African Institute for Development Policy
CQ: chloroquine
EvIDenCe: Evidence Informed Decision-making Centre
IPTp-SP: intermittent preventive treatment of malaria during pregnancy with sulfadoxine-pyrimethamine
KT: knowledge translation
KTP: Knowledge Translation Platform
MoH: Ministry of Health
NHRA: National Health Research Agenda
NMCP: National Malaria Control Programme
SP: sulfadoxine-pyrimethamine
